# Effect of alteplase on the CT hyperdense artery sign and outcome after ischemic stroke

**DOI:** 10.1212/WNL.0000000000002236

**Published:** 2016-01-12

**Authors:** Grant Mair, Rüdiger von Kummer, Zoe Morris, Anders von Heijne, Nick Bradey, Lesley Cala, André Peeters, Andrew J. Farrall, Alessandro Adami, Gillian Potter, Geoff Cohen, Peter A.G. Sandercock, Richard I. Lindley, Joanna M. Wardlaw, For the IST-3 Collaborative Group

**Affiliations:** From the Division of Neuroimaging Sciences (G.M., Z.M., A.J.F., G.C., J.M.W.) and the Division of Clinical Neurosciences (P.A.G.S.), University of Edinburgh, UK; the Department of Neuroradiology (R.v.K.), Dresden University Stroke Centre, Germany; Danderyd Hospital (A.v.H.), Stockholm, Sweden; Neuroradiology (N.B.), James Cook University Hospital, Middlesborough, UK; School of Pathology and Laboratory Medicine (L.C.), University of Western Australia, Perth; Cliniques Universitaires St Luc (A.P.), Neurologie, Belgium; Stroke Center (A.A.), Sacro Cuore-Don Calabria Hospital, Negrar, Italy; the Department of Neuroradiology (G.P.), Salford Royal NHS Foundation Trust, Manchester, UK; and the Westmead Hospital Clinical School and The George Institute for Global Health (R.I.L.), University of Sydney, Australia.

## Abstract

**Objective:**

To investigate whether the location and extent of the CT hyperdense artery sign (HAS) at presentation affects response to IV alteplase in the randomized controlled Third International Stroke Trial (IST-3).

**Methods:**

All prerandomization and follow-up (24–48 hours) CT brain scans in IST-3 were assessed for HAS presence, location, and extent by masked raters. We assessed whether HAS grew, persisted, shrank, or disappeared at follow-up, the association with 6-month functional outcome, and effect of alteplase. IST-3 is registered (ISRCTN25765518).

**Results:**

HAS presence (vs absence) independently predicted poor 6-month outcome (increased Oxford Handicap Scale [OHS]) on adjusted ordinal regression analysis (odds ratio [OR] 0.66, *p* < 0.001). Outcome was worse in patients with more (vs less) extensive HAS (OR 0.61, *p* = 0.027) but not in proximal (vs distal) HAS (*p* = 0.420). Increasing age was associated with more HAS growth at follow-up (OR 1.01, *p* = 0.013). Treatment with alteplase increased HAS shrinkage/disappearance at follow-up (OR 0.77, *p* = 0.006). There was no significant difference in HAS shrinkage with alteplase in proximal (vs distal) or more (vs less) extensive HAS (*p* = 0.516 and *p* = 0.580, respectively). There was no interaction between presence vs absence of HAS and benefit of alteplase on 6-month OHS (*p* = 0.167).

**Conclusions:**

IV alteplase promotes measurable reduction in HAS regardless of HAS location or extent. Alteplase increased independence at 6 months in patients with and without HAS.

**Classification of evidence:**

This study provides Class I evidence that for patients within 6 hours of ischemic stroke with a CT hyperdense artery sign, IV alteplase reduced intra-arterial hyperdense thrombus.

Arterial hyperattenuation on noncontrast CT, the hyperdense artery sign (HAS), is a consistently recognized CT sign of acute ischemic stroke.^1^ HAS is highly specific and moderately sensitive for intracranial arterial obstruction by thrombus.^2^ HAS is associated with increased stroke severity at presentation and worse long-term outcomes.^3–5^ There are, however, limited data on how the location, extent, or persistence of HAS relates to functional outcome following stroke, and importantly whether patients with (vs without) HAS benefit differently from IV thrombolysis with alteplase.^6–9^

The Third International Stroke Trial (IST-3) was a large (n = 3,035), multicenter, randomized controlled trial testing IV alteplase given within 6 hours of ischemic stroke.^10^ A central masked panel assessed prerandomization and follow-up CT for the presence of HAS.

We analyzed IST-3 imaging data to investigate whether, in this large, prospectively studied group of patients, the presence, location, and extent of HAS was associated with response to IV alteplase assessed as both change in HAS on short-term follow-up and also its effect on 6-month functional outcome.

## METHODS

### IST-3.

IST-3 was an international, multicenter, prospective, randomized, open, blinded endpoint (PROBE) trial of IV alteplase in acute ischemic stroke. Enrollment, data collection, image analysis, and CONSORT compliance have been described elsewhere.^10,11^ Briefly, adult patients with acute stroke of any severity, with no upper age limit, were eligible if IV alteplase could be started within 6 hours of stroke onset and CT or MRI had excluded intracranial hemorrhage and any structural stroke mimic. IST-3 used the uncertainty principle for enrollment, i.e., if the randomizing clinician believed that alteplase was clearly indicated or contraindicated, such a patient could not be enrolled; patients were only enrolled when there was genuine uncertainty over the benefit of alteplase for that individual.^10,11^ Stroke severity prior to randomization was assessed with the NIH Stroke Scale (NIHSS). After entry of baseline data, patients were randomized to receive IV alteplase (0.9 mg/kg) or control. Results were analyzed on an intention-to-treat basis. Patients were followed by postal questionnaire or masked telephone interview at 6 months and functional status assessed with the Oxford Handicap Scale (OHS).

### Standard protocol approvals, registrations, and patient consents.

Ethical approval for IST-3 was granted by the Scotland A research ethics committee and by local ethics committees. Informed consent was obtained for all patients. IST-3 is registered with Current Controlled Trials, ISRCTN25765518.

### Imaging protocol.

The IST-3 imaging protocol has been described previously.^10,12^ Briefly, CT scans were required to cover the brain from foramen magnum to vertex, with maximum slice thickness 4–5 mm through the posterior fossa and 8–10 mm for the cerebral hemispheres, with no slice gap. Thinner slices were also accepted. Scans were windowed on 80 Hounsfield units (HU) width and center level of 35–40 HU. Follow-up brain imaging (CT or MRI) was also required, to the same protocol, for all patients, between 24 and 48 hours after stroke onset. All imaging was reviewed centrally for quality control and validation.

### Image analysis.

A centralized panel of neuroradiologists and neurologists experienced in reviewing stroke imaging analyzed all imaging with an online assessment tool, the Systematic Image Review System (SIRS), recording assessments on a validated structured pro forma^1,13^ available at www.sbirc.ed.ac.uk/research/imageanalysis.html, accessed November 23, 2015. Assessors were masked to all other imaging and clinical data. HAS presence was determined visually (i.e., the reader decided if a vessel appeared hyperdense; no objective HU measurements were made). Where present, the location and extent of HAS was recorded by selecting the 3 largest vessels affected from the following options: internal carotid artery (ICA), middle cerebral artery (MCA) mainstem, sylvian branches of MCA, anterior cerebral artery (ACA), posterior cerebral artery (PCA), vertebral artery, basilar artery. A subgroup of scans (n = 272) underwent a second independent read as part of the IST-3 angiography and perfusion imaging substudy.^14^ Secondary reads were performed by a different panel of assessors (see coinvestigator list on the *Neurology*® Web site at Neurology.org) using SIRS as described above. Scans that were read twice were used to test interrater reliability of HAS assessment.

### Data analysis.

These analyses are restricted to patients with a noncontrast CT obtained prerandomization. The location of HAS was classified as proximal (ICA, MCA mainstem, vertebral or basilar arteries) or distal (ACA, PCA, or sylvian branches of the MCA). HAS extent was classified by the number of contiguous named vessels involved (0, 1, 2, or 3 as per the predefined options). We compared prerandomization and follow-up scans in all patients who had noncontrast CT performed at both times and calculated the change in HAS segment number from a minimum of −3 to a maximum of +3 (negative numbers indicate shrinkage and positive numbers indicate growth of HAS).

### Statistics.

We used univariate and multivariate tests to examine differences between all IST-3 patients and the subset who were HAS-positive prerandomization, between the treatment and control groups with HAS, and for associations among the presence, extent, location, and persistence of HAS, baseline clinical features, and effect of alteplase.

For univariate analysis of parametric data, we used *t* tests to compare ratios and means; for nonparametric data, we used Mann-Whitney *U* tests. We calculated χ^2^ statistics for dichotomous data. We used Krippendorff α (K-α) to test interrater reliability. K-α results range from −1.0 to +1.0 where +1.0 equates to perfect agreement, 0.0 means no agreement, and −1.0 implies perfect disagreement.^15^

We used multivariate ordinal regression to calculate common odds ratios (ORs) with 6-month OHS and change in HAS segment number as dependent variables.^16^ We tested the effect of HAS presence, extent, and location on outcome in separate models. Consistent with the main IST-3 analyses,^10,17^ multivariate models were adjusted for the effect of patient age, NIHSS, and time from stroke onset to prerandomization scan since these variables predicted outcome in the main trial.^10^ To reduce confounding between HAS extent and location on outcome, the effect of location was assessed only among those with HAS in a single arterial segment. To stabilize ordinal regression estimates, we used the same approach as the original IST-3 analysis of functional outcome, where the more severe grades of OHS (4–6) were grouped as one, leaving 5 ordinal analysis groups.^17^ Similarly, the variable time from stroke onset to prerandomization scan was grouped into 6 1-hour windows (0–6 hours), the variable time from prerandomization scan to follow-up scan was grouped into 5 12-hour windows (≤12, 13–24, 25–36, 37–48, and >48 hours) and the variable change in HAS segment number was grouped into 3 outcomes (fewer segments = shrinkage, no change, more segments = growth).

We performed tests of interaction (using Comprehensive Meta-Analysis software; Biostat, Englewood, NJ) to compare ORs for the effect of IV alteplase on change in HAS extent in distal vs proximal HAS and in 1 segment vs >1 segment HAS and to compare the effect of alteplase on outcome in those with vs those without HAS.

All other analyses were performed with IBM SPSS statistics software, version 20.0 (IBM Corporation, Armonk, NY). We considered *p* < 0.05 significant.

### Primary research question.

To assess the response of HAS to IV alteplase and to determine if the presence, location, extent, or persistence of HAS modified the effect of alteplase on outcome.

### Classification of evidence.

This study provides Class I evidence that IV alteplase (0.9 mg/kg) given within 6 hours of ischemic stroke onset accelerates shrinkage of HAS at follow-up 24–48 hours later.

## RESULTS

### Demographics and clinical and outcome data.

Most IST-3 patients had noncontrast CT performed both prerandomization (2,961/3,035, 97.6%) and at follow-up (2,779/3,035, 91.6%) 24–48 hours later. MRI was the imaging method in 56 and 151 patients, respectively. Prerandomization or follow-up CT scans were not available for central review in 18 (0.6%) and 105 cases (3.5%), respectively. Thus, the total number of patients with centrally reviewed CT both prerandomization and at follow-up was 2,731 (90.0% of 3,035). HAS data were missing for one follow-up CT (poor quality). There were no significant differences in the demographic and clinical data for the 2,961 patients with centrally reviewed prerandomization CT, nor for the 2,731 patients who also had follow-up CT when both groups were compared with 3,035 patients in the full IST-3 trial (data not shown).

The results of interrater reliability are as follows: for HAS identification, K-α = 0.40; for assessment of the largest artery involved, K-α = 0.46; and for assessment of HAS extent, K-α = 0.39.

Of 2,961 patients with prerandomization CT, 716 (24.2%) demonstrated HAS. On univariate analysis, patients with baseline HAS were younger and had a more severe stroke and worse 6-month outcomes than those without HAS (table 1). Among patients with HAS, those allocated control had an increased median time between prerandomization and follow-up scans compared with patients allocated alteplase. None of the other demographic or clinical measures in patients with HAS were significantly different between alteplase and control groups (table e-1).

**Table 1:**
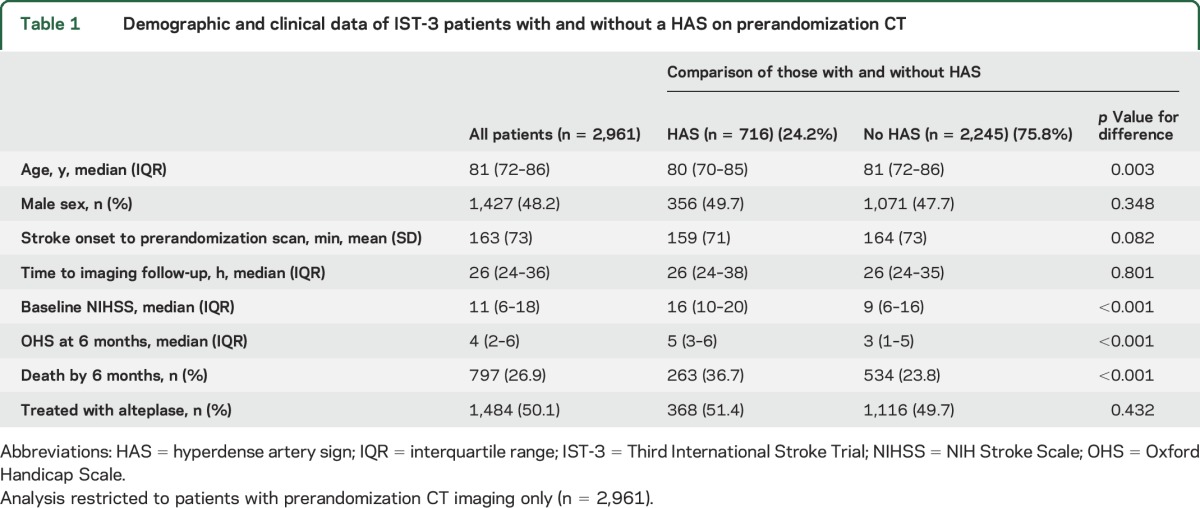
Demographic and clinical data of IST-3 patients with and without a HAS on prerandomization CT

**Figure 1:**
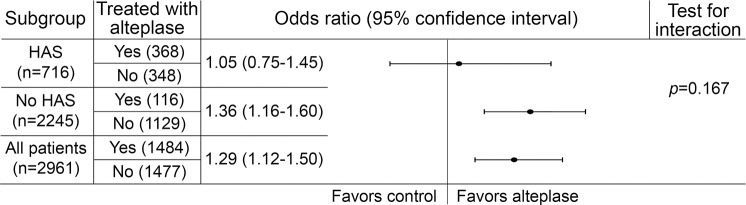
Separate ordinal regression analyses show odds ratios for the effect of alteplase treatment on 6-month functional outcome in the full group (n = 2,961) and in the subgroups with and without a hyperdense artery sign (HAS) on prerandomization CT Odds ratio >1 (right of line) indicates better outcome (lower 6-month Oxford Handicap Scale). Results for the HAS and no HAS groups are adjusted for the effect of age, time from stroke onset to scan (hours), and NIH Stroke Scale (NIHSS) score. Results for the full group are adjusted for the effect of age, time from stroke onset to scan (hours), NIHSS, and presence/absence of HAS.

For the multivariable ordinal analysis testing prerandomization variables associated with 6-month outcome in the whole group (OHS as dependent variable, n = 2,961), alteplase treatment allocation independently predicted a better outcome (lower OHS); OR 1.29, 95% confidence interval (CI) 1.12–1.50, *p* = 0.001. In the same analysis, the presence vs absence of HAS independently predicted a worse outcome (higher OHS, figure e-1); OR 0.66, 95% CI 0.55–0.80, *p* < 0.001. These results are adjusted for age, NIHSS, and time from stroke onset to scan (table 2 and figure 1).

**Table 2:**
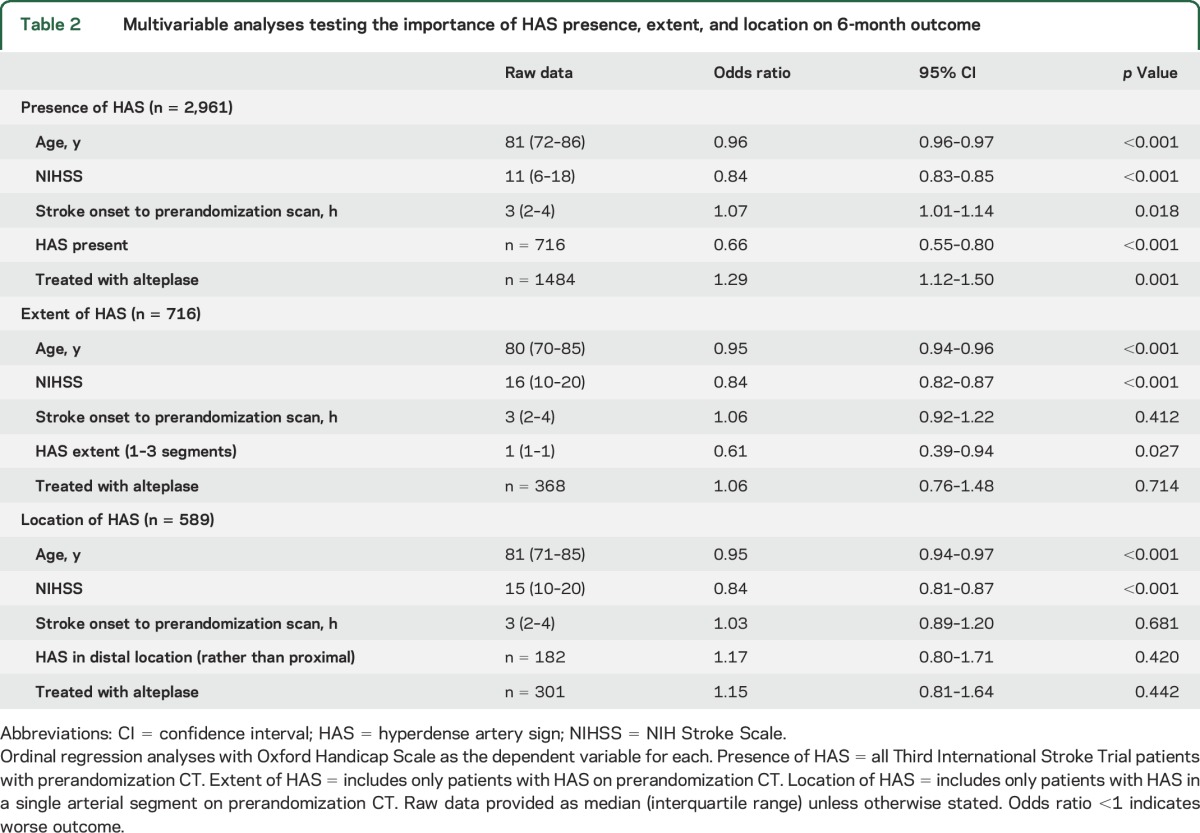
Multivariable analyses testing the importance of HAS presence, extent, and location on 6-month outcome

### Location, extent, and persistence of HAS.

For 2,731 patients with CT performed prerandomization and at follow-up, HAS was identified on 674 (24.7% of 2,731) prerandomization and 520 (19.0%) follow-up scans. In 870 cases (31.9%), HAS was evident on at least one scan. On univariate analyses, 6-month outcome was worse if HAS was found only in proximal rather than only in distal vessels, if HAS was more vs less extensive, if HAS showed growth rather than shrinkage between scans, if HAS persisted at follow-up rather than disappeared, and if a new HAS developed between scans (table e-2).

For multivariate analyses testing the effect of HAS characteristics on outcome (OHS as dependent variable) among patients with HAS in the whole group (n = 716), HAS extent (1, 2, or 3 segments) independently predicted a worse outcome (higher OHS); OR 0.61, 95% CI 0.39–0.94, *p* = 0.027. In patients with HAS in only 1 arterial segment (n = 589), HAS location (proximal vs distal) was not independently associated with outcome, OR 1.17, 95% CI 0.80–1.71, *p* = 0.420. These analyses are adjusted for age, NIHSS, time from stroke onset to scan, and treatment allocation (table 2).

### Effect of alteplase on HAS.

There were univariate associations between alteplase treatment and an increased likelihood of both HAS shrinkage and HAS disappearance (222/440 = 50.5% vs 166/429 = 38.7% shrank, χ^2^ = 12.6, *p* = 0.002, and 198/350 = 56.6% vs 151/323 = 46.7% disappeared, χ^2^ = 6.5, *p* = 0.011, respectively). In patients treated with alteplase, distal vs proximal HAS (65/86 = 75.6% vs 105/198 = 53.0%, respectively, χ^2^ = 12.7, *p* < 0.001) and single segment vs multisegment HAS (170/284 = 59.9% vs 28/66 = 42.4%, respectively, χ^2^ = 6.6, *p* = 0.010) were more likely to have disappeared at follow-up. Fewer patients who received alteplase had developed a new HAS in the interim between pre-randomization and follow-up CT, although the difference was not significant (90/957 = 9.4% vs 106/904 = 11.8%, χ^2^ = 2.2, *p* = 0.143).

**Figure 2:**
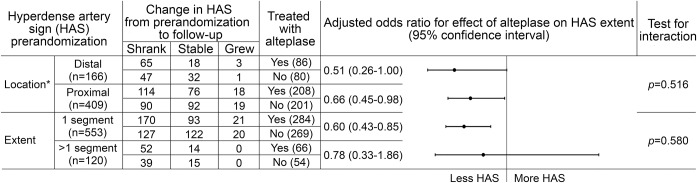
Ordinal regression analyses for the effect of treatment on change in HAS extent from prerandomization to follow-up scan Ordinal regression analyses with change in HAS segment number (shrinkage, no change, growth) as the dependent variable assessing the importance of prerandomization HAS location (proximal = internal carotid artery, middle cerebral artery mainstem, vertebral or basilar arteries; distal = anterior or posterior cerebral arteries or sylvian branches of the middle cerebral artery) and HAS extent on the effect of alteplase. *Location analysis does not include patients with HAS in both proximal and distal arteries. Odds ratio <1 (left of line) indicates HAS shrinkage, >1 (right of line) indicates HAS growth. Results are adjusted for the effect of age, time from stroke onset to scan (hours), and NIH Stroke Scale score.

For multivariate analyses, testing factors associated with change in HAS segment number (dependent variable), alteplase treatment was an independent predictor of HAS shrinkage at follow-up (OR 0.77, 95% CI 0.65–0.93, *p* = 0.006), while HAS growth was more likely in older patients (OR 1.01, 1.00–1.02, *p* = 0.013) (table 3). Both proximal and distal HAS were equally likely to shrink following alteplase treatment (OR 0.66, 95% CI 0.45–0.98, and OR 0.51, 95% CI 0.26–1.00, respectively), with no evidence of an interaction between HAS location and alteplase effect (*p* = 0.516) (figure 2). HAS affecting a single arterial segment prerandomization was more likely to shrink following alteplase treatment (OR 0.60, 0.43–0.85), but alteplase was not an independent predictor of HAS shrinkage if more than 1 segment was affected prerandomization (OR 0.78, 95% CI 0.33–1.86). Nevertheless, there was no evidence of an interaction between HAS extent and alteplase effect (*p* = 0.580) (figure 2). These results are adjusted for patient age, time (between prerandomization and follow-up scans), and stroke severity.

**Table 3:**
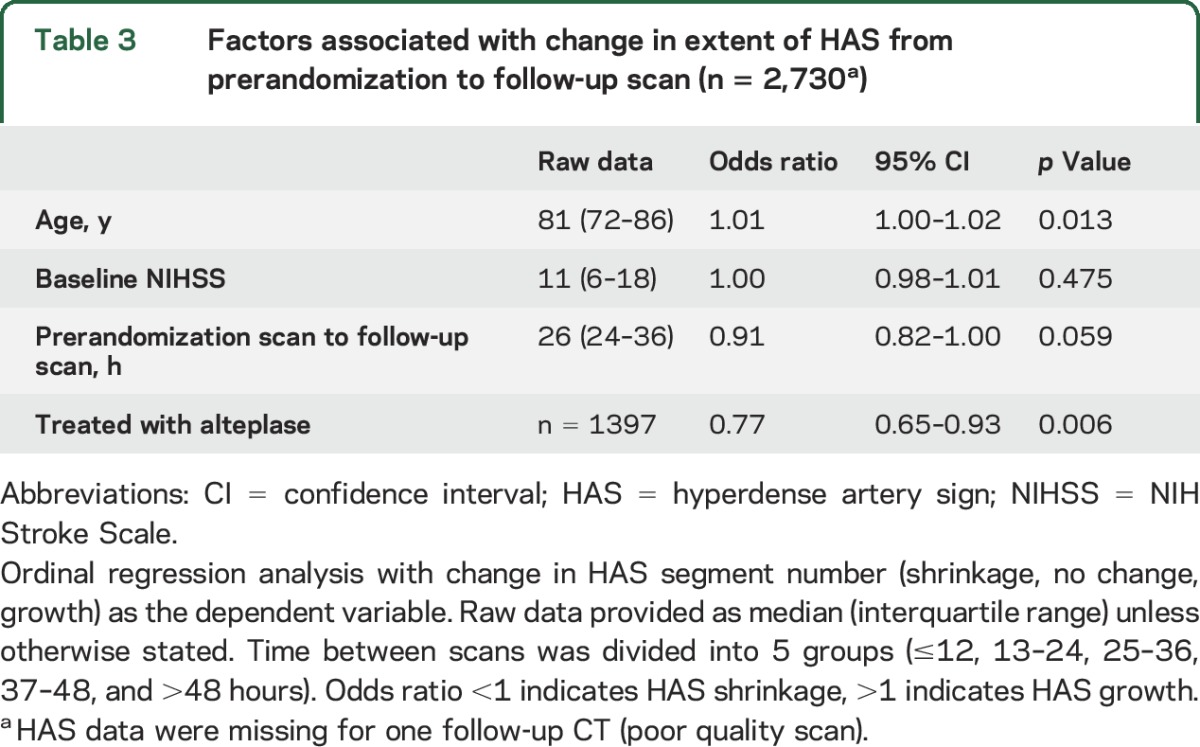
Factors associated with change in extent of HAS from prerandomization to follow-up scan (n = 2,730^a^)

### Effect of alteplase on patient outcome in those with HAS.

Results of multivariate analysis of alteplase effect on outcome (OHS as dependent variable) for the subgroups with (OR 1.05, 0.75–1.45) and without (OR 1.36, 1.16–1.60) HAS on prerandomization CT are shown in figure 1. There was no evidence of an interaction with the presence vs absence of HAS (*p* = 0.167).

## DISCUSSION

In this analysis of IST-3, we show that IV alteplase both reduces the persistence (more likely to shrink, or disappear) and limits the formation (less likely to grow) of HAS compared to patients allocated control. We found that alteplase increases HAS shrinkage/disappearance independent of age, baseline stroke severity, and time between prerandomization and follow-up scans. While HAS disappearance following treatment with alteplase was more common for small or distal vs large or proximal HAS on univariate analysis, the alteplase effect on HAS reduction/shrinkage was similar across all subgroups on multivariate regression analysis. We found no difference in the likelihood of HAS shrinkage for proximal vs distal HAS or for single segment vs multisegment HAS, i.e., alteplase reduces all HAS but is less able to remove the larger volume completely, usually more proximally sited HAS. We should note that alteplase treatment did not independently predict reduction of multisegment HAS but there was a trend toward such an association and this analysis is underpowered. Our findings are particularly relevant given recent evidence supporting the use of endovascular clot retrieval in ischemic stroke^18–20^ since not all patients will have the opportunity to receive, or be eligible for, such treatment. In addition, we and others^7,8^ have shown improved patient outcome where HAS shrinks or disappears with IV alteplase. Conversely, increasing patient age was an independent predictor of HAS growth, which may help to explain poorer stroke outcomes in the elderly.

Our work demonstrates a novel association between IV thrombolysis and measurable growth/shrinkage of HAS using randomized controlled trial data. We found no evidence that presence or absence of HAS materially altered the benefit of alteplase (the favorable shift in 6-month OHS). While the ordinal regression results for the HAS group alone are nonsignificant, this subgroup is underpowered and should be interpreted with caution. The appropriate interpretation is that there is no evidence of a difference in treatment effect between those with and without HAS at baseline, not that alteplase has no effect in those with HAS. To understand the apparent benefit of alteplase in patients without HAS, the presence of undetected thrombosis may be an answer; a recent report demonstrated benefit from thrombolysis for patients without apparent arterial obstruction on angiography.^21^

In IST-3, HAS was present in approximately 25% of patients within 6 hours of acute stroke. This is consistent with other published large series using similar scanning protocols^22,23^ although HAS prevalence rates of 47%–59% were reported in smaller studies.^24,25^ We confirmed that patients with HAS have more severe strokes and worse 6-month outcomes than patients without HAS. The effect of HAS presence on outcome is independent of age, time from stroke onset, stroke severity, and treatment with IV alteplase, i.e., patients with and without HAS both benefit from alteplase. Other authors have demonstrated that HAS independently predicts outcome,^8,26,27^ but our work also demonstrates that more extensive HAS independently predicts worse outcome among patients with the sign. We did not demonstrate an independent association between HAS location and outcome despite highly significant univariate results as demonstrated elsewhere^9^; however, our multivariate analysis of outcome by HAS location is limited by the exclusion of those HAS affecting more than one arterial segment and may be underpowered.

Our study has limitations. First, some CT scans were not thin-section but prevalence rates for HAS are greater with thin-slice protocols.^2^ Our prerandomization HAS prevalence may therefore be an underestimate. Second, our estimates of interrater reliability ranged from K-α 0.39 to 0.46. Prior studies have reported kappa (numerically equivalent to K-α) for the identification of HAS in the 0.36–0.91 range, but these studies rarely involved more than 2 readers, whereas our panel consisted of 10 individuals masked to correlative clinical and imaging data.^2,28^ Third, we did not define HAS based on HU measurements. Our central image analysis was performed qualitatively to reflect acute stroke care. A recent cohort study demonstrated that following IV alteplase, ischemic stroke patients with persistent arterial occlusion had lower mean density of thrombus compared to those who achieved recanalization.^29^ We are assessing the effect of thrombus density on outcome in IST-3 in separate analyses. Fourth, we estimated hyperdensity extent based on the number of arterial segments affected. A volumetric measurement may have provided a more accurate assessment of changes in HAS volume. However, it remains unclear if HAS volume can be measured reliably. Our method of assessing thrombus extent is relevant to daily practice. Fifth, we used HAS disappearance as a surrogate indicator of arterial recanalization in the absence of angiographic data for all patients. We are analyzing available angiography data from IST-3 in a separate analysis. Sixth, multivariate models were adjusted only for variables that were associated with outcome in the main IST-3 analysis^10^ and for the variable time from stroke onset to scan since this latter variable would likely affect the appearance of HAS. Seventh, the limitations of the full IST-3 trial have been previously discussed^10^ including use of the uncertainty principle for enrolment and the potential introduction of bias through the adoption of an open design. Finally, even with 3,000 patients, IST-3 has insufficient power to reliably explore interactions between HAS and the effect of alteplase, but we found little evidence that patients with HAS responded differently to alteplase than those without HAS.

We confirmed, using data from a large randomized controlled trial, associations between the presence and extent of HAS and poor 6-month outcomes. We show clearly that alteplase accelerates shrinkage of HAS, regardless of HAS location and extent. Furthermore, we found no evidence that the improvement in functional outcome following alteplase was materially different in the presence or absence of HAS. Therefore, while HAS is useful to support a diagnosis of acute ischemic stroke and helps predict outcome, our data suggest that the presence or absence of the sign should not preclude the use of IV alteplase where clinically appropriate.

## Supplementary Material

Data Supplement

Coinvestigators

## GLOSSARY

ACA: anterior cerebral artery
CI: confidence interval
HAS: hyperdense artery sign
HU: Hounsfield units
ICA: internal carotid artery
IST-3: Third International Stroke Trial
MCA: middle cerebral artery
NIHSS: NIH Stroke Scale
OHS: Oxford Handicap Scale
OR: odds ratio
PCA: posterior cerebral artery
SIRS: Systematic Image Review System
