# Supplementary material for: Effect of alteplase on the CT hyperdense artery sign and outcome after ischemic stroke
Source: Neurology. 2016 Jan 12;86(2):118–25. doi: 10.1212/WNL.0000000000002236 (PMC4731690; doi:10.1212/WNL.0000000000002236)
Supplement: Data Supplement [file supp_86_2_118__index.html]

Data Supplement 

# Effect of alteplase on the CT hyperdense artery sign and outcome after ischemic stroke

## Data Supplement

Two tables and one figure; one PDF file.

**Neurology® data supplements are not copyedited before publication. Published editorials and translations have been copyedited.  
 © 2016 American Academy of Neurology.  
  
 Files in this Data Supplement:**

- Tables e-1 to e-2, Figure e-1 - PDF file
