# Supplementary material for: Effect of alteplase on the CT hyperdense artery sign and outcome after ischemic stroke
Source: Neurology. 2016 Jan 12;86(2):118–25. doi: 10.1212/WNL.0000000000002236 (PMC4731690; doi:10.1212/WNL.0000000000002236)
Supplement: Data Supplement [file supp_WNL.0000000000002236_Supplemental_data.pdf]

Table e-1. Demographic and clinical characteristics for patients with HAS on pre-randomization CT (n=716) comparing those treated with alteplase and those allocated control

	**Treated with Alteplase n=368** (51.4%)	**Allocated Control n=348** (48.6%)	**p-value for Difference**
**Age** (median, IQR)	80 years (71-85)	80 years (69-85)	0.891
**Male sex**	180 (48.9%)	176 (50.6%)	0.657
**Stroke onset to pre-randomization scan** (mean, SD)	161 minutes (71)	157 minutes (70)	0.451
**Time to imaging follow-up** (median, IQR)	26 hours (23-34)	27 hours (24-41)	0.004
**Baseline NIHSS** (median, IQR)	16 (10-20)	16 (10-20)	0.843
**OHS at six months** (median, IQR)	5 (3-6)	5 (3-6)	0.646
**Death by six months**	136 (37.0%)	127 (36.5%)	0.898

Footnote:

Results represent n (%) unless otherwise stated.

NIHSS = National Institutes of Health Stroke Scale. OHS = Oxford Handicap Scale. IQR = Interquartile Range. SD = standard deviation.

Table e-2. Univariate associations between imaging characteristics of HAS on baseline and follow-up CT and six-month outcome (OHS)

Hyperdense Artery Sign (HAS) Characteristics on CT		n	Median OHS (IQR)	p-value for Difference
**HAS Location Pre-Randomization**	Proximal vessel only	407	5 (3-6)	$p < 0.001$
	Distal vessel only	182	4 (2-6)	
**HAS Extent Pre-Randomization**	1 segment	589	5 (3-6)	$p = 0.009$
	2-3 segments	127	5 (4-6)	
**Pre-Randomization to Follow-Up Change**	Growth	237	5 (3-6)	$p < 0.001$
	Shrinkage	388	4 (2-6)	
**Persistence to Follow-Up**	Persisted	324	5 (3-6)	$p < 0.001$
	Disappeared	349	4 (2-6)	
**New HAS at Follow-Up**	Yes	196	5 (3-6)	$p < 0.001$
	No	1861	3 (1-5)	

Footnote:

Total numbers in univariate analyses vary because proximal/distal data does not include patients with HAS in both proximal *and* distal arteries and growth/shrinkage data does not include patients where HAS extent remained unchanged between scans.

OHS = Oxford Handicap Scale. IQR = Interquartile Range.

Follow-up CT was only available in 674 of those with and in 2057 of those without HAS at baseline.

HAS data were not available for one follow-up CT.

Figure e-1. Effect of hyperdense artery sign (HAS) pre-randomization on six-month outcome

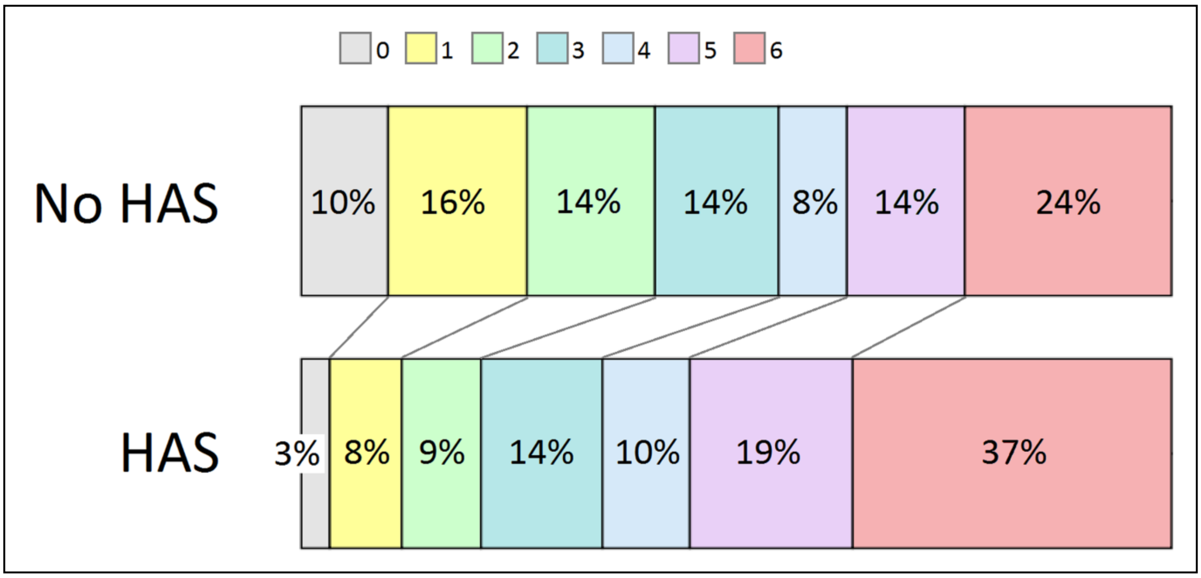

Footnote:

Ordinal regression analysis for six-month Oxford Handicap Scale (OHS, 0-6) comparing outcome in those with (n=716) and without (n=2245) a Hyperdense Artery Sign (HAS) on pre-randomization CT.

Independent effect for the presence of HAS was Odds Ratio = 0.66 (<1 indicates worse outcome), 95%CI = 0.55-0.80, p<0.001 (see Table 2a for full result of this regression analysis). Result adjusted for the effect of age, time from stroke onset to scan, National Institutes of Health Stroke Scale (NIHSS) and treatment allocation.
