# Supplementary material for: Effect of alteplase on the CT hyperdense artery sign and outcome after ischemic stroke
Source: Neurology. 2016 Jan 12;86(2):118–25. doi: 10.1212/WNL.0000000000002236 (PMC4731690; doi:10.1212/WNL.0000000000002236)
Supplement: Coinvestigators [file supp_WNL.0000000000002236_Coinvestigators_R.docx]

**Co-Investigator Appendix.** IST-3 Collaborative Group

For a complete list of all committees, please see the IST-3 primary publication in The Lancet (The benefits and harms of intravenous thrombolysis with recombinant tissue plasminogen activator within 6 h of acute ischaemic stroke (the third international stroke trial [IST-3]): a randomized controlled trial. *Lancet* 2012;379:2352-63).

**CO-INVESTIGATORS**

Peter Sandercock, DM (University of Edinburgh, Scotland, co-chief Investigator); Richard I Lindley, MD (Sydney Medical School – Westmead Hospital and The George Institute for Global Health, University of Sydney, Australia, co-chief Investigator); and Joanna M Wardlaw, MD (University of Edinburgh, Scotland, co-chief Investigator for neuroradiology).

**CONTRIBUTORS**

**Trial Steering Committee**

Colin Baigent (University of Oxford, UK, independent chairman); David Chadwick (University of Liverpool, UK, independent chairman); Pippa Tyrrell (University of Manchester, UK, independent member); Gordon Lowe (University of Glasgow, UK, independent member); Martin Dennis (University of Edinburgh, Scotland); Geoff Cohen (University of Edinburgh, Scotland, statistician); Karen Innes (University of Edinburgh, Scotland, trial coordinator); Heather Goodare (lay representative).

**National coordinators and associate national coordinators**

Australia: Richard I Lindley, MD (Sydney Medical School – Westmead Hospital and The George Institute for Global Health, University of Sydney, Australia); Graeme J Hankey (Royal Perth Hospital, Perth). Austria: Karl Matz (Landesklinikum Donauregion Tulln, Tulln), Michael Brainin. Belgium: AP. Canada: Gord Gubitz (Dalhousie University and Queen Elizabeth II Health Sciences Centre, Halifax), Stephen J Phillips (Dalhousie University and Queen Elizabeth II Health Sciences Centre, Halifax). Italy: Stefano Ricci (Department of Neurology ASL1, Ospedale, Citta’ di Castello). Mexico: Antonio Arauz (Instituto Nacional de Neurologia, Mexico City). Norway: Eivind Berge (Oslo University Hospital, Oslo), Karsten Bruins Slot (Oslo University Hospital, Oslo). Poland: Anna Czlonkowska (Institute of Psychiatry and Neurology, Warsaw, and Medical University of Warsaw, Warsaw), Adam Kobayashi (Institute of Psychiatry and Neurology, Warsaw, Poland). Portugal: Manuel Correia (Hospital Geral de Santo Antonio, Porto). Switzerland: Phillippe Lyrer (University Hospital Basel, Basel), Stefan Engelter. Sweden: Veronica Murray (Karolinska Institutet, Stockholm), Andreas Terent, Bo Norrving, Per Wester: UK: Graham Venables (Sheffield Teaching Hospitals NHS Foundation Trust, Sheffield, UK).

**CT and MRI reading panel**

Joanna M Wardlaw, Andrew Farrall (University of Edinburgh, Scotland), Zoe Morris (University of Edinburgh, Scotland), Rüdiger von Kummer (Dresden University Stroke Centre, Germany), Lesley Cala (University of Western Australia, Crawley, Australia), Anders von Heijne (Dandyred Hospital, Stockholm, Sweden ), Alessandro Adami (Sacro Cuore-Don Calabria Hospital, Verona, Italy), Andre Peeters (Cliniques Universitaires Saint-Luc, Bruxelles, Belgium), Gillian Potter (Salford Royal NHS Foundation Trust, England), Nick Bradey (Neuroradiology, James Cook University Hospital, South Tees Hospital NHS Trust, Middlesborough, UK).

**Angiography reading panel**

Joanna M Wardlaw, Rüdiger von Kummer, Andrew Farrall, Robin Sellar (University of Edinburgh, Scotland), Alessandro Adami, Philip White (Newcastle University, UK), Andrew Demchuk (University of Calgary, Canada), Matthew Adams (Great Ormond Street Hospital, London, UK), Grant Mair (University of Edinburgh, Scotland), Bernard Yan (The Royal Melbourne Hospital, Parkville, Australia).

**IST-3 collaborative group and participating hospitals in each country**

Figures in parentheses are the number of patients recruited in the country or by the centre.

***UK*** *(1447)* Royal Hallamshire Hospital (118): G Venables, C Blank, H Bowler, C Doyle, K Endean, K Harkness, E Parker, M Randall. University Hospital of North Staffordshire (97): C Roffe, N Ahmad, A Arora, S Brammer, J Chembala, B Davies, S Ellis, E Epstein, K Finney, C Jackson, C Jadun, R Kinston, H Maguire, I Memon, I Natarajan, M Poulson, R Sanyal, S Sills, A Vreeburg, E Ward. Western General Hospital (95): P Sandercock, R Al-Shahi Salman, R Davenport, M Dennis, P Hand, S Hart, I Kane, S Keir, M MacLeod, L McKinlay, H Milligan, E Sandeman, J Stone, C Sudlow, P Taylor, J Wardlaw, C Warlow, W Whiteley, A Williams. The National Hospital for Neurology & Neurosurgery (84): M Brown, B Athwal, V Bassan, N Bhupathiraju, J Bowler, C Davie, D Doig, R Erande, S Gilbert, L Ginsberg, R Greenwood, S Gregoire, N Harding, N Losseff, R Luder, N Passeron, R Perry, P Rayson, R Simister, S Stone, D Werring. Arrowe Park Hospital (83): J Barrett, H Aitken, S Cherian, R Davis, S Downham, L Godd, V Gott, D Jose, V Little, D Lowe, L Luxford, M McGrory, P Owings, N Price, J Richards, G Sangster, J Sherlock, S Vargese, I Wakefield, P Weir. Southend University Hospital (77): P Guyler, T Attygale, S Chandler, L Coward, S Feasey, C Khuoge, T Loganathan, S Martin, A O'Brien, D Sinha, V Thompson, S Tysoe, R Walsh. Norfolk and Norwich University Hospital (67): K Metcalf, J Cochius, R Fulcher, N Gange, C Green, J Jagger, M Lee, P Myint, J Potter, G Ravenhill, S Shields, N Shinh, T Staunton, E Thomas, W Woodward, P Worth, N Wyatt. Nottingham City Hospital (63): W Sunman, P Bath, P Berman, J Clarke, C Gaynor, F Hammonds, R Harwood, K Mitchell, S Munshi, S Pacey, A Shetty, N Sprigg, H Stear, G Subramanian, A Wills. Guy's & St.Thomas Hospital (60): A Rudd, H Audebert, A Bhalla, J Birns, R Chowdhury, G Cluckie, I Davies, C Gibbs, P Holmes, N Mitchell, F Schiavone, E White, M Yeung. Darlington and Bishop Auckland Hospitals (56): A Mehrzad, V Baliga, E Brown, L Burnside, B Esisi, J Kent, P Orr, D Stead, E Wayman. University Hospital Aintree (46): R Durairaj, C Cullen, R Kumar, H Martin, D McDowell, A Sharma, V Sutton, R White. University Hospital of Wales (46): T Hughes, K Ali, J Anderson, K Baker, K Bethune, K Bethune, M Booth, M Cossburn, S Halpin, M Hourihan, E Marsh, K Peall, R Powell, H Shetty, M Wardle, M Williams. Derby Royal Hospital (37): K Muhiddin, J Beavan, M Clarke, R Donneley, S Elliott, P Fox, P Gorman, M Harper, M Mangoyana, I Memon, L Mills, L Wright. Addenbrookes Hospital (34): L Warburton, J Baron, P Barry, D Day, T Harold, P Martin, J Mitchell, E O'Brien, J Rycarte, M Turnham. St George's Healthcare NHS Trust (34): G Cloud, L Choy, B Clarke, C Griffin, O Halse, I Jones, F Kennedy, U Khan, R Lewis, A Loosemore, C Lovelock, H Markus, B Moynihan, J O'Reilly, O Paul, A Pereira, M Punter, P Rich, D Rolfe, F Schiavone. Royal Devon & Exeter Hospital (Wonford) (30): M James, J Bell, A Bowring, L Boxall, J Cageao, H Eastwood, S Elyas, F Hall, S Harries, A Hemsley, S Jackson, S Keenan, P Mudd, A Sekhar, D Strain, J Sword, N Wedge. Aberdeen Royal Infirmary (26): M MacLeod, M Bruce, A Joyson, M Kemp, K McMullan, J Reid, O Robb, J Webster, S Wilkinson. Hammersmith Hospitals & Imperial College (24): P Sharma, P Bentley, H Jenkins, A Kar, T Sachs. Northwick Park Hospital (20): D Cohen, R Bathula, J Devine, M Mpelembue. William Harvey Hospital (20): D Hargroves, I Balogun, L Cowie, A Maidment, D Rand, J Rowe, H Rudenko, D Smithard, L Wray. Scarborough Hospital (17): J Paterson, J Brown, J Hampton, S Jamieson, R Rose, A Volans. Countess of Chester Hospital NHS Foundation Trust (17): K Chatterjee, G Abbott, R Brookes, C Castle, C Kelly, S Leason, A Nallasivan, A Sen. Watford General Hospital (17): D Collas, M Cottle, N Damani, P Jacob, D Oza, D Werring. University Hospitals Coventry & Warwickshire NHS Trust (15): A Kenton, N Adab, L Aldridge, H Allroggen, Y Brown, R Cross, L Galvin, K Ghosh, A Grubneac, A Lindahl, H Mehta, M Pritchard, C Randall, P Ray, A Shehu, S Thelwell. Royal Bournemouth & Christchurch NHS Trust (12): D Jenkinson, J Bell, T Black, O David, J Kwan, A Orpen, C Ovington, D Tiwari, Z ud Din Babar. Leeds General Infirmary (12): A Hassan, A Bailey, J Bamford, C Bedford, R Bellfield, J Cooper, L Dunsmure, J Greig, M Keeling, L Mandizvidza, J Rankine, E Roberts, P Wanklyn, T Webb, S Williamson. York Health Services NHS Trust (12): J Coyle, S Crane, C Croser, P Duffey, R Evans, E Iveson, M Keeling, G Kitching, M Porte, C Rhymes. Queen Elizabeth Hospital (Gateshead) (12): D Barer, M Armstrong, M Bokhari, T Cassidy, B McClelland. Queen Elizabeth The Queen Mother Hospital (10): G Gunathilagan, P DOLKE, S Jain, S Jones, A Maidment, L Rosser, G Thomas, C White. Worcestershire Royal Hospital (10): P Sanmuganathan, C Scholtz, E Stratford. Blackpool Victoria Hospital (10): M O'Donnell, H Goddard, G Hoadley, J Howard, S Leach, J McIlmoyle, A Stewart, A Strain. Basildon & Thurrock University Hospitals NHS FT (9): F Huwez, P Croot, N Gadi, N Mguni, U Umasankar. Royal Infirmary of Edinburgh (8): G Mead, B Chapman, A Coull, S Hart, A Kinnear, B Morrow, F Morrow. St Mary's Hospital (8): D Ames, J Ball, S Bannerjee, J Chataway. Yeovil District Hospital (8): K Rashed, C Buckley, D Donaldson, D Hayward, C Lawson. Luton and Dunstable Hospital (8): L Sekaran, K Bharaj, F Justin, G Jutlla, D Phiri, S Sethuraman, M Tate. Solihull Hospital, Heart of England NHS Trust (8): D Sandler, P Carr, G Jones, J Lyons, K Warren. King's College Hospital (7): L Kalra, A Davis, J Jarosz, D Manawadu, L Sztriha. Doncaster Royal Infirmary (7): D Chadha, A Holford, P Willcoxson. Royal United Hospital Bath (7): L Shaw, D Button, A Cunningham, L Dow, J Dutson, T Hall, C Hardy, N Jakeman, P Kaye, B Madigan, K O'Brien, D Pressdee, M Price, L Robinson, C Taylor, D Williamson. Birmingham Heartlands Hospital (6): D Sandler, P Carr, J Lyons, J McCormack, C Stretton. University Hospital North Durham (6): P Earnshaw, E Brown, S Bruce, C Church, S Desai, B Esisi, M Myint, N Watt. Wansbeck General Hospital (6): C Price, S Elliott, H Graham, R Lakey, K Mitchelson. Bristol Royal Infirmary (6): P Murphy, L Ball, S Caine, J Dovey, J Hughes, A Steele. Stepping Hill Hospital (6): K Dizayee, A Brown, T Chattopadhyay, J Cheetham, H Cochrane, A Datta, M Datta-chaudhuri, C Fox, D Kilroy, S Krishnamoorthy, F Levy, S Metha, P Ngoma, B Venkatesh. Princess Royal Hospital Brighton & Sussex University Hospitals Trust (5): K Ali, R Gautam, N Henderson, M Jones, S Murphy, G Spurling. Belfast City Hospital (5): I Wiggam, C Boyd, K Fullerton, P Gray, M Kinnaird, S MacNair, C Morgan, M Reid, S Tauro. Royal Liverpool University Hospital (5): S Loharuka, D Balmforth, P Cox, G Fletcher, A Ledger, A Manoj, M Wilkinson. City Hospital, Sandwell & West Birmingham Hospital (5): D Nicholl, S Clegg, S Hurdowar, S Kausar, K Law, A Singal, S Sturman. Royal London Hospital (4): P Gompertz, J Evanson, A Farrell, A Petrou, K Saastamoinen, T Sachs, A Salek-Haddadi, R Yadava. Sunderland Royal Hospital (4): J O'Connell, H Brew, S Butler, S Crawford, C Gray, D Gulliver, N Majmudar, R O'Brien. Morriston Hospital (4): M Wani, L Dacey, L Davies, R Evans, D Harris, T Jones, S Storton. Royal Preston Hospital (4): S Punekar, A Ashton, S Duberley, H Emsley, C Gilmour, B Gregary, L Hough, S Philip, S Wuppalapati. The Royal Wolverhampton Hospitals NHS Trust (4): K Fotherby, P Bourke, D D'Costa, K Kauldhar, D Leung, R Lodwick, S McBride, D Morgan, M Qaiyum, G Sahota, M Srinvasan. Royal West Sussex NHS Trust, St Richard's Hospital (4): I Kane, N Chuter, L Garrad, M Hookway, S Ivatts, G Kennedy. Queen's Hospital Romford (4): K Darawil, L Al Dhahirl, S Andole, M Baig, P Dugh, K Dunne, H Kariuki, M Khan, S Rathnayaka. Ulster Hospital (3): M Power, K Dynan, J Finnerty, A Heaney, C Leonard, K McKnight, J Turkington, B Wroath. Great Western Hospital (3): B Dewan, S Cotton, M Gardiner, T Saunders, B Vincent. The Queen Elizabeth Hospital Birmingham (3): D Sims, P Guest, E Jones, J McCormack, D Nicholl, J Savanhu, R Tongue, M Willmot. Leicester General Hospital (3): D Eveson, S Dawson, M Dickens, M Fotherby, R Hunt, S Khan, T Kumar, R Marsh, A Mistri, T Robinson, J Thompson. Darent Valley Hospital, Dartford & Gravesham NHS Trust (3): P Aghoram, T Daniel, M Gatehouse, S Hussein, A Jackson, T Shanganya, E Strachan, G Tan. Nevill Hall Hospital, Aneurin Bevan Local Health (3): B Richard, S Elaine, S Hanson, S Mosely, H Reed, M Williams. Colchester Hospital University Foundation Trust (3): R Saksena, S Cook, D Demuran, M Keating, R Needle, V Paramsothy, A Sebastian, R Sivakumar, A Wright. Salford Royal Hospital Foundation NHS Trust (2): R Grue, E Barberan, C Dickson, C Douglas, J Jellicoe, T Marsden, J Priestley, E Quick, C Sherrington, A Singh, C Smith, J Stevens, P Tyrell, J Wainwright. Leicester Royal Infirmary (2): M Ardron, J Birchall. Queen Elizabeth Hospital (Kings Lynn) (2): R Shekhar, C Barsted, S Coleman, S Fletcher, J Graham. John Radcliffe Hospital (2): A Buchan, J Hinkle, J Kennedy, A Manoj, M Westwood. Derriford Hospital (2): A Mohd Nor, S Allder, B Hyams, A Pace. West Cumberland Hospital (1): E Orugun, C Brewer, L Huntley, R Jolly, C Summers. Sandwell General Hospital (1): K Sharobeem, J Khaira, J Leahy, E Linehan, G Moore, J Rizkalla, J Wilkinson. Torbay Hospital (1): D Kelly, C Hilaire. Warrington & Halton Hospitals NHS Foundation Trust (1): O Otaiku, L Connell, G Delaney-Sagar, G James, L Lomax, D Matthew, J Simpson, H Whittle. Medway Maritime Hospital (1): S Sanmuganathan, S Burrows, A Mahmood. Southampton General Hospital (1): G Durward, S Barker, J Cantle, P Crawford, S Evans, V Pressly, N Weir. Victoria Hospital (1): V Cvoro, K McCormick. ***Poland*** *(347)* 2^nd^ Department of Neurology, Institute of Psychiatry & Neurology (190): A Czlonkowska, J Bembenek, M Bilik, G Chabik, W Czepiel, J Dzierka, M Gluszkiewicz, K Grabska, B Janus-Laszuk, J Jedrzejewska, A Kobayashi, T Litwin, A Oskedra, A Piorkowska, M Skowronska, A Sliwinska, U Stepien. SPZZOZ w Sandomierzu (43): P Sobolewski, A Gajewska, M Grzesik, R Hatalska-Zerebiec, I Labudzka, B Loch, A Medrykowska, M Sledzinska, A Sobota, W Szczuchniak, G Wolak, I Zdyb. Medical University of Gdansk (35): W Nyka, D Gasecki, K Chwojnicki, A Gojska, B Karaszewski, G Kozera, M Kwarciany, M Nowak, M Swierkocka-Miastkowska , S Szczyrba, M Wisniewska, E Wnorowska. 1^st^ Department of Neurologyt, Institute of Psychiatry & Neurology (25): P Richter, A Bochynska, M Chahwan, A Graban, R Rola. Military Medical Institute (24): A Stepien, B Brodacki, M Grotowska, J Kotowicz, J Staszewski, J Swistak, S Zaloga. Szpital Powiatowy (14): J Stoiñski, K Czajkowaka-Fornal, P Czubak, A Kaczor, J Kraska, E Nowakowska-Sledz, J Ozdoba-Rot, E Zawadzka. Szpital Specjalistyczny w Konskich (8): M Fudala, D Adamczyk, W Brola, I Guldzinska, K Kaluzny, M Kucharska-Lipowska, M Mosiolek, M Polewczyk, M Ziomek. Central University Hospital (7): G Opala, M Arkuszewski, M Kudlacik, P Malgorzata, M Swiat. SPSK im. Prof. W. Orlowskiego CMKP (1): U Fiszer, M Lenska-Mieciek. ***Italy*** *(326)* Ospedale Di Citta' di Castello (62): S Cenciarelli, A Barilaro, R Condurso, F Coppola, S Dioguardi, E Gallinella, A Mattioni, C Menichetti, S Ricci. Nuovo Ospedale Civile "S.Agostino-Estense" (40): F Casoni, M Bacchelli, M Cavazzuti, M Malagoli, A Zini. Ospedale Beato Giacomo Villa - Citta' della Pieve (36): G Benemio, M Celani, R Allegrucci, V Bondo, S Cupella, L Guerra, S Guerrieri, C Ottaviani, E Righetti, C Rossi, N Sacchi, M Scucchi, V Stefanini. Ospedale Di Branca (36): T Mazzoli, A Bigaroni, L Greco, R Paris, P Parise, S Ricci. A.O. Niguarda Ca'Granda (25): A Ciccone, L Basso, R Causarano, P Doneda, E Ferrante, A Gatti, A Guccione, A Gullo, F Imbesi, S Jann, R Marazzi, E Moro, C Motto, D Parodi , A Protti, M Riva, A Rosiello, I Santilli, R Sterzi, P Tiraboschi, G Venturelli. R.Guzzardi Hospital - Vittoria (RG) (19): F Iemolo, R Campagna, G Campagnolo, A Carnemolla, N D'Apico, G D'Asta, S Giannarita, A Giordano, E Sanzar. Ospedale Regionale di Aosta (17): E Bottacchi, S Cordera, G Corso, M Di Giovanni, G Giardini, C Lia, T Meloni, M Pesenti Campagnoni, P Tosi. Ospedale Sacro Cuore - Negrar Verona (12): A Adami, G Rossato, T Zuppini. IRCCS Istituto delle Scienze Neurologiche, Ospedale Maggiore - Bologna (12): G Procaccianti, T Sacquegna. Universita degli Studi di Genova (11): C Gandolfo, M Balestrino, C Bruno, L Castellan, M Del Sette, A Ferrari, C Finocchi, N Reale, D Rizzi. Ospedale Civlle S.Andrea (9): M Del Sette, L Benedetti, C Capellini, E Carabelli, E Cibei, M Godani, G Guariglia, E Landini, A Mannironi, B Nucciarone, S Parodi, S Tonelli, E Traverso, D Zito. Ospedale S. Giovanni Battista - Foligno (8): P Brustenghi, F Corea, O Flamini, S Lolli, G Pelliccia, R Ricci, S Stefanucci, M Zampolini. Ospedale a Vibo Valentia (8): D Consoli, F Galati, P Postorino. Azienda Ospedaliero-Universitaria "Ospedali Riuniti" di Foggia (8): G Rinaldi, E Carapelle, G Grilli, M Guido, L Specchio. Ospedale Valduce di Como (8): N Checcarelli, G Borin, L Chiveri, R Clerici, E Corengia, L Gandola, P Garavaglia, M Guidotti, A Martegani, M Mauri, F Muscia, F Raudino. Ospedale di Cattinara - Trieste (4): F Chiodo Grandi, A Bratina, N Carraro, M Gaio, A Granato, N Koscica, M Naccarato, V Sarra, P Schincariol, C Vilotti, Z Zugna. Clinica Dr Pederzoli Spa (4): D Idone, C Bonato, E De Angelis, A Forgione, M Gambera, F Recchia, S Tamburin, P Tinazzi Martini, G Zanette. Ospedale Civile San Matteo Degli Infermi - Spoleto (2): S Grasselli. Ospedale Silvestrini - Perugia (2): G Agnelli, A Andrea, A Billecia, V Caso, V Casso, R Fabiola, P Fanelli, M Paciaroni, B Sergio, M Vemti. Mater Salutis Hospital, Legnago VR (2): M Silvestri, L Altarini, A Bonfante, M Bonornetti, B Costa, N D'Attoma, N Deluca, F Frattini, R Niego, D Rafaele, V Ravenna, M Turazzini. Ospedale Guglielmo da Saliceto - Piacenza (1): S Cammarata. ***Sweden*** *(297)* Uppsala University Hospital (100): E Lundström, L Jonsson , U Söderström, A Terént. Danderyd Hospital (46): V Murray, A Alvelius, M Arbin von, I Dalenbring, Å Doverhall, Å Franzén-Dahlin, N Greilert, M Hallberg, A Heijne von, E Isaksson, H Kumpulainen, A Laska, A Lundström, C Martin, J Muhrbeck, E Näslund, N Ringart, E Rooth, R Undén, P Waldenström. Hassleholm Hospital (29): M Esbjornsson, M Petranek. Karnsjukhuset (25): B Cederin, E Bertholds, A Elgåsen, T Johansson, B Witteborn. Koping Hospital (20): M Kwiatkowska, E Gustafsson, T Noren, J Saaf. Mora Hospital (17): J Teichert, M Bertilsson, S Nilsson, S Oestberg. LidkopingHospital (11): L Welin, K Fredricson, L Pehn. Falu Hospital (11): J Hambraeus, I Lonn. Capio S: tGoran Hospital (9): B Hojeberg, A Adolfsson, M Anzen. Vastervik Hospital (5): T Wallen, R Schloenzig, P Söderström, A Wennerberg. University Hospital MAS (5): F Buchwald, K Abul-Kasim, A Berkeskold, J Petersson, E Poromaa. University Hospital of Northern Sweden (4): P Wester, R Backlund, A Sjöström. Helsingborgs lasarett (4): B Hedström , E Campbell, K Johnsson, B Karlsson, N Lekokotla, C Lundahl, A Risedal, P Sandgren, A Svensson. Visby Hospital (4): S Bysell, E Smedberg, A Vestberg Bysell. Sundsvall Hospital (3): V Sjögren, B Högvall. University Hospital Lund (2): G Andsberg, T Cronberg, A Lindgren. Vasteras Hospital (1): H Wannberg, F Ax, L Nyren. Karlstad Central Hospital (1): J Sanner, H Andersson, F Andler, S Holmgård, R Johansson, I Magnussan, K Nilsson, J Rådberg. ***Norway*** *(204)* Trondheim University Hospital (69): B Indredavik, H Ellekjær, A Østvik, G Rohweder, D Steckhan, J Storvold. Oslo University Hospital Sykehus (66): E Berge, Y Rønning, R Aakvik, K Bruins Slot, G Knutsen, M Moxness, R Pettersen, T Wyller. University Hospital of North Norway (23): C Wahl, O Iversen, S Johnsen, B Norderhus, L Steffensen, E Stensland. Kongsvinger Hospital (13): T Asak, J Aaseth, T Rotnes, J Sparby, S Wetterhus. Levanger Hospital (9): H Hallan, A Aardal, T Graven, H Hansbakk Skjetne, B Klykken, K Lindqvist, A Tommy. University Hospital of North Norway (8): T Engstad, M Antonsen, R Bajic, W Fønnebø, S Hykkerud, I Lyngmo, A Nyrnes, S Rogne, S Sparr. Harstad Hospital (7): O Kildahl-Andersen, K Pedersen, H Ulrichsen. Ålesund Hospital (4): O Skogen, I Alnes, R Hukari, Y Seljeseth, P Vadset.  Asker and Bærum Hospital (2): G Knutsen, B Fure, H Ihle-Hansen, N Johnsen, L Kornberg. Namsos Hospital (2): S Schuler, M Heibert. Volda Hospital (1): M Lillebø, O Aasen, I Eskeland, T Hamre, S Hareide, H Helset, K Kolnes, B Lødemel, H Ose Velle, S Reite, E Velle. ***Australia*** *(179)* Nambour General Hospital (51): R Grimley, E Ahern, C Cocks, M Courtney, R Devin, J Endacott , C Fawcett , V Harrington, C Johnston, M Koltermann, S Murray, K Ng, G Styles, A Tampiyappa. John Hunter Hospital (29): C Levi, K Chung, L Dark, M Evans, Y Gawarikar, E Kerr, A Loiselle, F Miteff, A Moore, W O'Brien, M Parsons, D Quain, A Royan, M Russell, N Spratt. Gosford Hospital (24): J Sturm, D Crimmins, D Griffiths, P Kavelieros, J Kinsella, A Malhotra, B O'Brien, A Schutz, M Webb, S Whyte, V Zenteno. Westmead Hospital (16): R Lindley, A Bleasel, N Cordato, A Duggins, V Fung, L Gomes, N Ingham, J Ip, P Landau, J Morris, S Vucic. Royal Perth Hospital (15): G Hankey, A Claxton, N Lillywhite. The Canberra Hospital (12): C Lueck, C Andrews, G Danta, C Das, I Harvey, A Hughes, C McColl, A Oon, R Tuck. Royal Brisbane and Women's Hospital (10): S Read, M Badve , M Broad, G Cadigan, H Cavanagh, J Chalk, D Copsinis, K Etherington, R Henderson, R Hull, J O'Sullivan, J Pandian, L Ross-Lee, M Roxas, N Sheikh, G Skinner, A Wong. Austin Health - Repatriation Campus (8): H Dewey, A Brodtmann, G Donnan, A Hughes, M Karonen, H Ma, T Mulcahy, S Petrolo, L Walker, D Young, J Zavala. Nepean Hospital (7): M Thieben, C Harris, M Krause, S Lane, H Park, M Shaffi, J Wood. Box Hill Hospital (7): C Bladin, A Buckland, K Coughlan, B Coulton, A Gilligan, P Lee, S Mullen, Z Ross, P Sien Loh, C Szoeke. ***Portugal*** *(82)* UAVC. Centro Hospitalar de Trás-os-Montes e Alto Douro (42): M Silva, F Afonso, J Gabriel, P Guimarães, A Velon. Hospital Pero da Covilhã (19): M Castelo- Branco, F Alvarez, V Branco, C Coxo, P Goulao, D Leal, S Morgado, R Oliveira, F Paiva, A Rodrigues, M Simoes. Hospital de Santo António (12): G Lopes, T Almeida, M Cardoso, J Chaves, C Correia, M Correia, J Damásio, R Felgueiras, J Pereira, A Tuna. Hospital S.Marcos (9): C Ferreira, E Lourenco, A Machado, R Mare, J Rocha. ***Belgium*** *(73)* Cliniques Universitaires St Luc (73): A Peeters. ***Austria*** *(46)* Landesklinikum Donauregion Tulln (34): K Matz, M Brainin, G Funk, V Reiner-Deitemyer. Krankenhaus Der Barmherzigen Bruder Wien (9): J Ferrari, A Flamm-Horak, G Gruber, R Rattinger. Krankenhaus Göttlicher Heiland (3): W Muellbacher, D Doppelbauer, R Kalchmayr, W Schima, T Wieser, M Zart. ***Switzerland*** *(23)* Universitatsspital Basel (22): P Lyrer, L Bonati, S Engelter, F Fluri, S Muller, E Radue, A Tiemessen, L Walz, F Weisskopf, S Wetzel. Universitätsspital Zürich (1): A Luft, D Fetz, B Hertler, A Pangalu. ***Canada*** *(8)* QEII Health Sciences Centre (8): G Gubitz, P Boulton, J Jarrett, J Moeller, S Phillips. ***Mexico*** *(3)* Instituto Nacional de Neurologia y Neurojrugia MVS (3): A Arauz, L Bermudez, J Calleja, R Garcia.
